# Comparative Research of Microstructure and Mechanical Properties of Stainless and Structural Steel Dissimilar Welds

**DOI:** 10.3390/ma14206180

**Published:** 2021-10-18

**Authors:** Saulius Baskutis, Jolanta Baskutiene, Regita Bendikiene, Antanas Ciuplys, Karolis Dutkus

**Affiliations:** 1 Department of Production Engineering, Faculty of Mechanical Engineering and Design, Kaunas University of Technology, Studentu Str. 56, LT-51424 Kaunas, Lithuania; saulius.baskutis@ktu.lt (S.B.); jolanta.baskutiene@ktu.lt (J.B.); antanas.ciuplys@ktu.lt (A.C.); 2 Umega Group AB, Kauno Str. 120, LT-20115 Ukmerge, Lithuania; karolis.dutkus@hennordic.com

**Keywords:** stainless steel, metal inert gas welding, dissimilar welds, microstructure, mechanical properties

## Abstract

The present study utilized a metal inert gas welding (MIG) to make a dissimilar weld of stainless steel AISI 304, 314, 316L, 420 grades and a standard structural steel S355MC. It refers to a weld joining two materials from different alloy systems commonly used in heat exchangers, pressure vessels, and power plant systems. Obviously, maintaining the integrity of such welds is of paramount importance to the safety issues. Therefore, detailed microscopic and experimental studies were performed to evaluate the reliability of these welds. The microscopic analysis did not reveal any presence of weld defects such as porosity or cracks, which ensured that MIG process parameters were properly selected. The performance of dissimilar welds was assessed by hardness and tensile tests. The hardness profiles revealed differences between austenitic and martensitic steel welds that later showed extremely high values in the heat-affected zone (HAZ), which caused fractures in this zone during tensile test. The welds of all austenitic steel grades withstood the tensile test, showing an average tensile strength of 472 MPa with fractures observed in the base metal zone. It made clear that the use of a filler rod 308LSI is suitable only for the austenitic stainless and structural steel dissimilar welds and not appropriate for martensitic-structural steel welds. The achieved results revealed that the higher hardness of the martensitic phase in the HAZ of AISI 420 is closely related with the formation of untempered coarse martensitic structure and higher carbon content.

## 1. Introduction

Generation of hybrid structures of materials provides huge opportunities for constructors in creating and developing new products with the required properties and high reliability. Requirements related to the welding procedure of dissimilar steel welds cover the production of boilers, tanks, heat exchangers, and pressure vessels used in various industries. Such a specific application requires different materials to be joined with high reliability and joint quality. However, due to the different complexity of materials, dissimilar welds may lead to unanticipated failures. The problems most commonly encountered in dissimilar weld joints are related to the formation of brittle phases and undesirable residual stress distributions across different zones of welds, which initiates formation of cracks or failures of the joint before the expected service life [1,2]. The majority of these negative consequences can be solved in principle by adjusting the microstructure while welding. In order to get a higher quality weld and to avoid defects in dissimilar welds, proper selection of welding metals, filler material (if used), and welding parameters are needed. The usage of filler ensures better control of the corrosion resistance and mechanical properties of weld [3]. Important factors influencing the strength of dissimilar metal weld are melting temperatures, thermal conductivity, coefficient of thermal expansion, dilution of metals in the weld area, and carbon migration from the steel having the higher carbon content toward lower carbon content steel [4]. In the welded joints with a similar base metal, the crack formation may be prevented by a subsequent post-weld heat treatment (PWHT) [5]. Unfortunately, the heat-affected zone (HAZ) of austenitic stainless steels remains unaffected even after PWHT, meanwhile the PWHT has a huge impact on the mechanical properties, the formation of the HAZ microstructure and a fusion zone (FZ) of carbon, and on martensitic alloyed steels [6]. The correlation of the fatigue strength, tensile properties, hardness, and microstructure of austenitic stainless and medium carbon steel dissimilar welds was reported [7] showing 40% lower fatigue strength of dissimilar welds compared to austenitic steel welds and 30% lower fatigue strength when compared to medium carbon steel welds. Dimensions of the fatigue process zone and the peculiarities of its formation were determined [8] by the method of laser interferometry and the optical method, and a three-dimensional finite element sub-model of the near-crack tip region for stress–strain state analysis of metal alloys at normal tension was developed [9].

Due to the fact that steels of the dissimilar welds possess different chemical composition, the major problem of microstructure heterogeneity arises [10]. This difference in the microstructure is a consequence of the formation of cracks not far from the fusion line, which is an undesirable factor and which increases the inner stress concentration in the adjacent HAZ. Free carbon movement in the dissimilar welds of 304 stainless and carbon steel [11] also influences crack formation. It was observed that because of small cracks, the weakest area between welds of dissimilar metals forms close to the fusion line or along the martensitic transition adjacent to the fusion line [12]. This phenomenon is caused by hydrogen absorbed during welding. The strength properties of dissimilar welded stainless and low-alloyed carbon steel are influenced by the diffusion of the base and weld metals, those having different coefficients of thermal expansion, which results in the different residual stresses across the various zones of weldments [13].

Martensitic stainless steels grades are used instead of austenitic ones when high strength and hardness are more easily achievable by heat treatment rather than by the cold working and when mechanical properties are more important than corrosion resistance. The welding of martensitic stainless steels may cause problems with increased hardness in the HAZ and the formation of martensitic structure with retained delta ferrite in it [1,14]. As it has been reported [14], the hardness of HAZ depends on the tempering temperature and is influenced by the production of more martensite to ferrite and carbide Me_23_C_6_ transformation, which decreases. The precipitation of Me_23_C_6_ carbide has a negative impact on both the mechanical properties and corrosion resistance of stainless steel AISI 420 weld. It was reported [15] that the increase in corrosion potential is greatly associated with a higher Cr content.

There are numerous studies on the mechanical properties’ investigation in similar welds of steels; however, there is limited information about the relation between mechanical properties and microstructure and on comparisons of dissimilar austenitic-structural and martensitic-structural steels welds.

This research was accomplished to evaluate the influence of the peculiarities of the microstructure on the tensile behavior and hardness of MIG welded dissimilar joints. The comparative evaluation was done in terms of the microstructure, hardness tests, and tensile test behavior. The chosen 3xx series of austenitic stainless steels and structural steel dissimilar welds present excellent cost and properties ratios, particularly in critical applications including oil and gas, the chemical industry, the pulp and paper industry, water systems, desalination plants, pollution control equipment, and chemical tankers. This series is often used when only certain parts of the welded structure require corrosion or heat resistance. Meanwhile martensitic stainless steel is generally selected for applications where a combination of high strength and corrosion resistance at ambient temperature is required. The absence of nickel and the lower content of other alloying elements in the latter steel makes them less costly than other stainless types. The main aim of this study was to understand the extent to which martensitic and structural steel joints can replace austenitic stainless steel and structural steel dissimilar welds using the same welding parameters and test conditions. Moreover, some of the achieved results were given as a guide to the welding practice of 3xx and 4xxx series and structural high-strength low-alloy steels’ dissimilar welds.

## 2. Experimental Procedure

The materials to be welded were 4-mm thick sheets of austenitic stainless steel grades AISI 304, 314, 316L, martensitic stainless steel AISI 420, and structural high-strength low-alloy steel S355MC. The martensitic stainless steel AISI 420 has relatively high hardness and high carbon content, whereas austenitic steels have a lower carbon but a higher chromium (up to 28%) content, and consequently a higher corrosion resistance. The details on the chemical composition and standardized mechanical properties of the steels under research are presented in Table 1 and Table 2, respectively.

**Figure 1:**
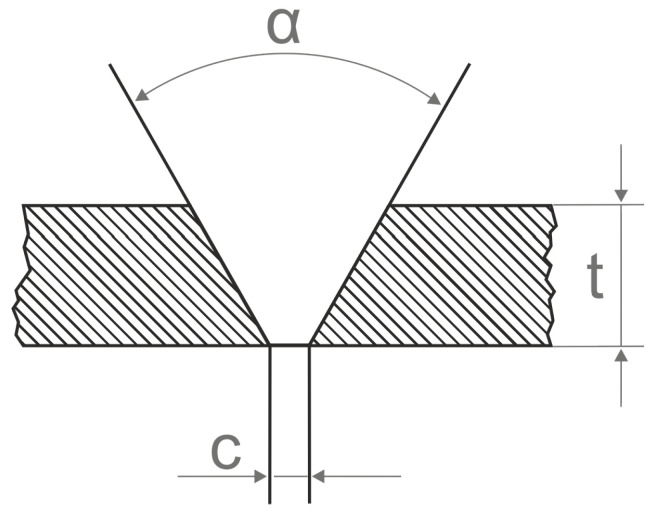
The geometry of sheet edge: α—an angle of the groove edge; t—the thickness of the weldment; c—root opening.

**Figure 2:**
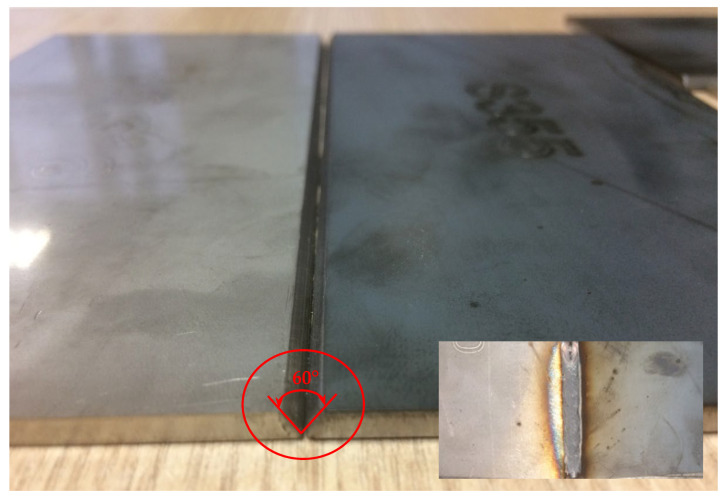
Grooved sheets of different steels’ grades prepared for welding; inlet after welding.

The welded sheets were prepared according to the recommendations specified in EN ISO 9692-1:2013 for the single-V butt weld, and the sheet edges with an angle of 60° were milled by a milling machine. The sheet edge geometry is presented in Figure 1. A 2-mm gap between the edges of the sheets was maintained. Before welding, all prepared samples (Figure 2) were polished and degreased with ethanol in order to remove any surface dirt, oxides, or dust.

The compact inverter welding machine Phoenix 355 Puls with an integrated wire feed mechanism was utilized to compose welded joints. The samples were arranged precisely in the welding machine. Welding was carefully accomplished in one pass along the groove using super-pulse and impulse welding techniques. The most suitable welding parameters were chosen according to the weld seem quality and are presented in Table 3.

A filler rod ER308LSi suitable for stainless steel MIG welding with a diameter of 0.8 mm was used (Table 1). This type of wire was chosen because of the minimum amount of carbon (the carbon content was held to a maximum of 0.02%) in the composition that allows reducing the possibility of inter-granular carbide precipitation and ensuring good resistance to general corrosion. The composition of filler rods usually follows the base metal composition; however, this is difficult to accomplish by welding different metals. Ni and Mn in the composition allow improving the toughness and strength of the weld, but these elements also lower the temperature of martensitic transformation, which in turn increases the risk of retained austenite formation.

**Figure 3:**
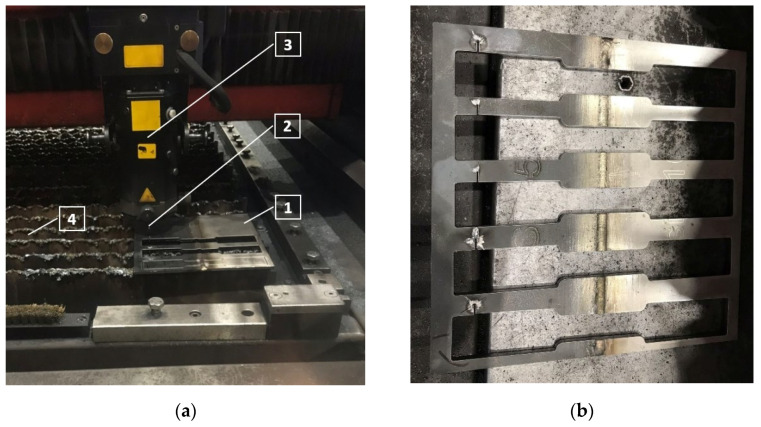
Laser cutting of the tensile samples: (**a**) cutting process with CNC laser-cutting machine: 1—workpiece; 2—nozzle; 3—laser cutting head; 4—workpiece supports; (**b**) dissimilar weld samples after laser cutting.

In order to prevent the liquid metal pool from the impact of the environment, pure argon (99.9%) was used as a shielding gas (flow rate 20 L/min), which ensures a wide and shallow penetration of the weld bead and enables alteration of the length of the metal arc, not changing the heat of the arc. When welding was completed, all the weld samples were cleaned and cut at a direction perpendicular to the weld into the test pieces for subsequent transverse tensile and hardness tests followed by the observation of microstructure. The cutting of the samples for tensile tests was done using 4 kW CNC laser-cutting machine Bystronic BySprint Fiber 3015 (Figure 3).

**Figure 4:**
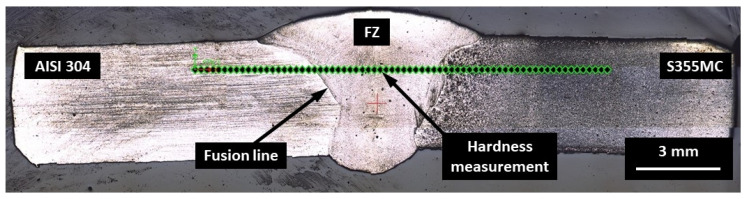
The cross section of the joint with the location of hardness test’s indentations.

The hardness test across the weld was executed using a Mitutoyo Hardness Testing Machine HM-200 (Mitutoyo Corporation, Kanagawa, Japan) using a diamond indenter under a load of 0.98 N [16], with a 10-s dwell time at 0.25-mm intervals 2 mm from the weld’s top surface, while the original hardness of the base metal (BM) was reached (Figure 4).

The transverse samples for optical analysis were prepared as required according to the basic procedures: grinding, polishing to near a mirror finish, following 30-s etching in Gliceregia etchant (15 mL HCl, 10 mL glycerol, and 5 mL HNO_3_). In order to distinguish the different zones of the dissimilar welds as well as to gain data on grain distribution and size, the examination of optical micrographs was done using an optical microscope Carl ZeisAxio Scope A1 with the set of the objectives ranging in linear magnification from 0.5× to 250×. The hardness tests were performed at the ambient temperature of 20 ± 2 °C under the relative humidity of 50 ± 5%.

**Figure 5:**
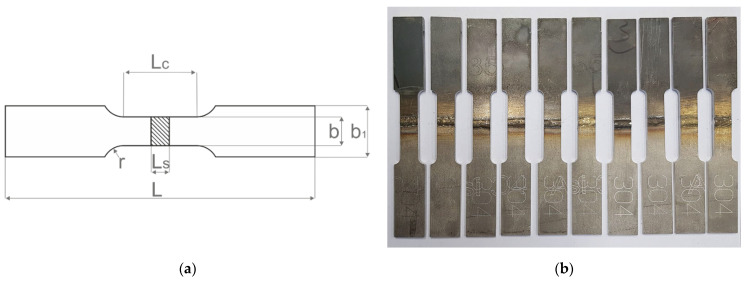
Tensile samples: (**a**) tensile sample geometry: L—total length; L_c_—gauge length; L_s_—thickness of sample; b and b_1_—width; (**b**) laser-cut samples prepared for testing.

The tensile test pieces were subjected to a transverse tensile test to evaluate the strength of the dissimilar weld and its exploitation properties [16]. The samples with a gauge length of 60 mm were prepared according to the ISO 4136:2012 standard as presented in Figure 5.

A 50-kN “Amsler” versatile electromechanical testing machine equipped with a HBM testing device was used to accomplish the tensile tests at a crosshead speed of 2 mm/min in the laboratory under the same conditions as the hardness tests.

## 3. Results and Discussion

### 3.1. The Analysis of Welds’ Microstructure

The microstructure of different zones in dissimilar weld joints was studied, i.e., in the filler metal-depleted zone (FZ); the partially melted zone (PMZ) (being observed close to the FZ, also known as dissolution zone); the HAZ, which is usually found as the weakest part in the weldment; and the BM as the area with no changes in the microstructure.

**Figure 6:**
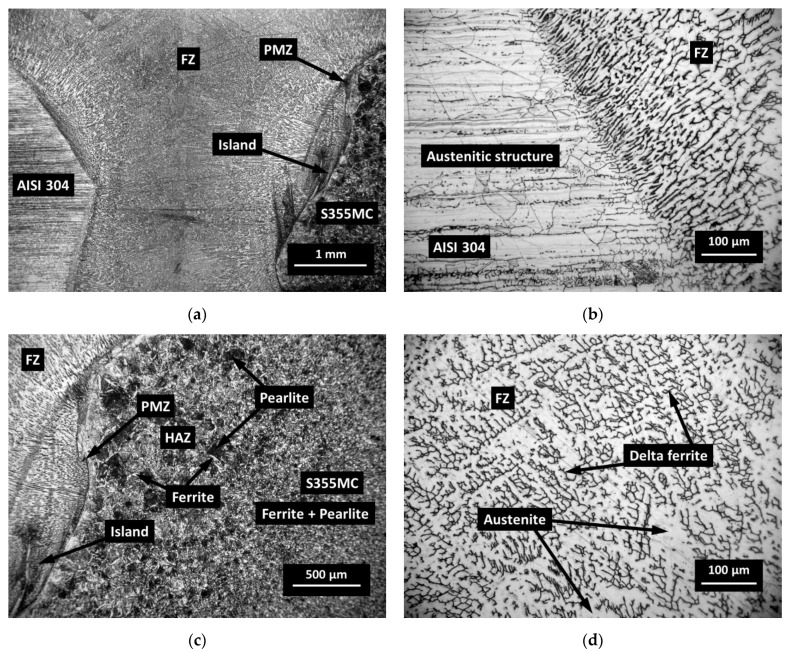
Optical images of different zones of welded sample AISI 304/S355MC: (**a**) general view; (**b**) partially melted zone AISI 304/FZ; (**c**) partially melted zone FZ/S355MC; (**d**) delta ferrite and austenite in FZ.

**Figure 7:**
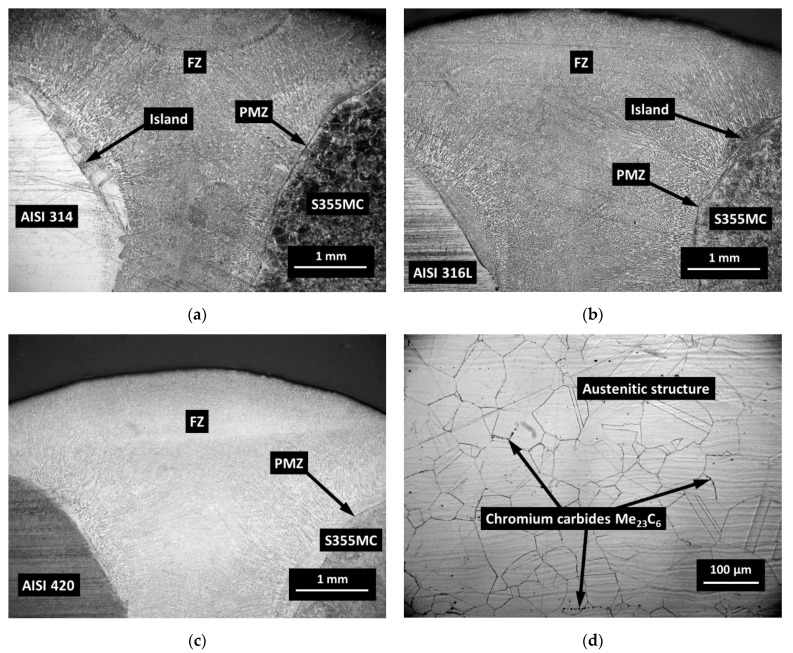
Optical images of dissimilar welds: (**a**) AISI 314/S355MC; (**b**) AISI 316L/S355MC; (**c**) AISI 420/S355MC; and (**d**) austenite grains in AISI 314.

The optical micrographs of the weld cross-section of dissimilar joint of AISI 304, 314, 316L, and 420 and structural steel S355MC are presented in Figure 6 and Figure 7. During the welding process due to the usage of a filler metal with a higher content of Cr, which has a great affinity to carbon, some of the interstitial mobile carbon atoms migrate from the PMZ [17]. This relatively narrow carbon depleted zone is called the decarburized region [18,19] or the carbon-depleted zone [20,21,22] (Figure 6a). It has been observed that at relatively high levels of chromium (24.19% in AISI 314), even a small carbon content can cause the formation of chromium carbides Me_23_C_6_ at the grain boundaries of austenitic grains, especially on slow cooling (Figure 7d). Each single fine Me_23_C_6_ carbide starts to grow, having a direct orientation to a matrix. Eventually, Me_23_C_6_ precipitates, forming a film-like coarse constituent, covering one adjacent grain side, and forming a low-Cr zone on the other adjacent grain side of the austenite grain boundary [23]. The presence of coarse Me_23_C_6_ carbides in a weldment has a negative effect on the mechanical properties. Therefore, during welding, apart from the appearance of carbide at the boundaries of austenitic grains, a local decrease in the content of chromium usually occurs because of short-term heating and slow cooling, which can cause steel affinity to an inter-granular corrosion. The increase in carbon content in the steel leads to a higher possibility of the chromium carbides precipitating at the grain boundaries.

The austenite phase in BM of stainless steels AISI 304 (Figure 6b), 314 (Figure 7d), and 316L is embedded in the ferrite matrix with an almost equal content of ferrite, while the microstructure of S355 MC steel consists of a mixture of perlite and acicular ferrite (Figure 6c) with a typical fine-grained and interlocking structure [24]. The low carbon content (0.049%) allows much more allotriomorphic ferrite to be formed with the grains that acquire an equiaxed form due to the effect of hard impingement [25]. Allotriomorphic means that the form of the ferrite does not reflect its internal crystalline symmetry. This can be explained by the fact that it grows faster along the surface of austenitic grains. As a result of this process, its contours adjust the γ grain boundaries. In the BM, the amount of pearlite slightly reduces due to the lower carbon content in the steel.

The FZ can be described as a mixture of fully molten BM and filler metal with a high degree of homogeneity where the mixing in the molten weld pool is primarily assured due to convection. As shown in Figure 6d, the austenite-based dendrites prevailed in the FZ. Additionally, optical micrographs of FZ (Figure 6d) showed the formed delta ferrite resulting from the ferrite-austenite solidification process. The highest delta ferrite content in the structure indicates that the weld is strong enough [26]. The increase in delta ferrite compared to the BM of AISI 304 was associated with relatively high temperature maintenance during the solidification process.

As an experimental study showed, the formation of dendritic carbides did not induce any brittleness in the FZ and did not impair the strength properties of the weld seam.

**Figure 8:**
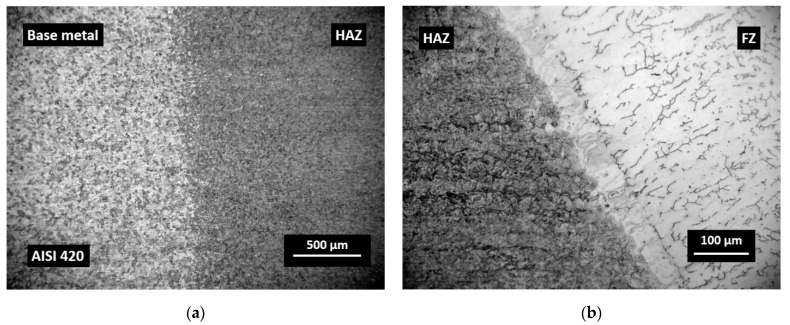
Optical images of AISI 420: (**a**) HAZ with coarse untempered martensite (right) and free tempered martensite in base metal (left); (**b**) partially melted zone AISI 420/FZ.

The optical micrographs of dissimilar weld AISI 420/S355MC and the microstructures across the HAZ from the FZ to AISI 420 BM are presented in Figure 7c and Figure 8, respectively.

These elongated fine crystals and relatively large inter-crystalline zones are the evidence of the typical dendritic structure (Figure 8b). Compared with the BM, the increase in austenite content in the weld area can be explained by the usage of filler road containing a relatively high content of nickel (10.2%). Examination of micrographs of dissimilar joints did not show the presence of weld defects such as porosity or cracks.

### 3.2. Mechanical Tests

The hardness and tensile tests were accomplished to evaluate the mechanical properties of the dissimilar welds.

**Figure 9:**
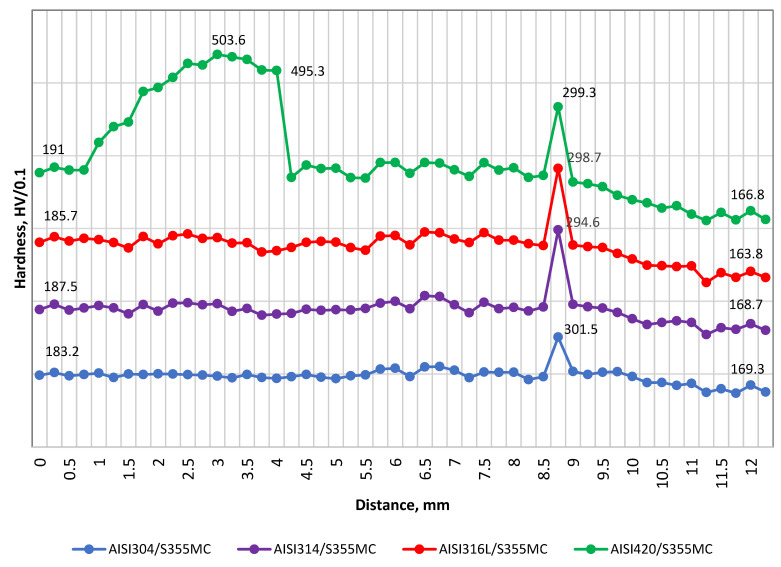
Hardness profile across the weld joints.

The hardness profile across the weld joints interface is presented in Figure 9. The hardness of the stainless steels is indicated on the left side and the hardness of steel S355MC on the right side. Apart from the peaks noticeable in the PMZ of the steel S355MC (~300 HV/0.1), no significant change in hardness was recorded in the stainless steels AISI 304, 314, and 316L except for the joint AISI 420 with S355MC (~500 HV/0.1) (Figure 9).

**Figure 10:**
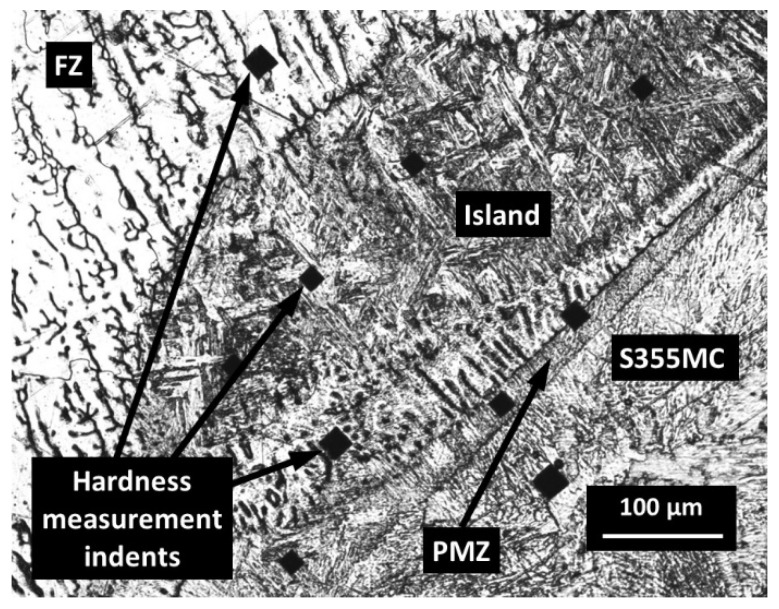
Microstructure of FZ/S355MC with the hardness test’s indentations.

The PMZ and HAZ areas of S355MC steel, affected by the temperature of austenitization during the welding process, were completely re-austenitized and then may subsequently transform to sorbite-troostite when slowly cooled until room temperature. A slower diffusion at lower temperatures resulted in the formation of a finer, harder, and stronger structure. In the PMZ area with a temperature of approximately 550 °C, the thickness of ferrite-cementite plates was just approximately 0.1 µm, and a structure known as troostite with a hardness of about 300 HV/0.1 was formed. Decreasing temperature influences the average reduction in austenite grain size with the accompanying decrease in hardenability. Thus, it can be stated that the hardness profile across the PMZ basically shows a peak of hardness at the FZ boundary with a gradual decrease across the coarse-grained HAZ. The peaks of the hardness with the width up to 50 µm were observed in the PMZ of steel S355MC to the FZ boundary and in the islands (Figure 6a,c and Figure 7a,b). Figure 10 presents the image of the weld with the hardness test indentations whose size clearly indicates the hardness. Shallow indentation on the island and PMZ indicates a higher hardness of these areas compared to the hardness of the FZ and HAZ of the base metal (299 HV/0.1 via 168 HV/0.1).

The significant increase in hardness of the stainless steel AISI 420 HAZ area (Figure 9) can be explained by the presence of fine untempered martensite that had a structure of small cementite particles in a fine-grained ferritic matrix that negatively affected the ductility [27]. Figure 8 shows the micrographs of the weld metal and HAZ resulting after the welding process: HAZ contained coarse untempered martensite, which was hard (~500 HV/0.1) and relatively brittle compared with the base metal, where hardness values dropped to ~190 HV/0.1, because the tempered martensite at first caused a decrease in hardness. The difference in hardness of HAZ and unaffected BM was ~62%.

**Figure 11:**
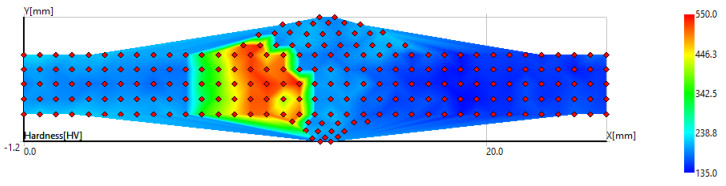
The map of hardness indentations of AISI 420/S355MC dissimilar weld.

The map of hardness showing the distribution of hardness values throughout the surface of the dissimilar weld AISI 420/S355MC is presented in Figure 11. This map allows to quantify the material properties along the length of microstructurally significant weld zones [28]. The employed hardness mapping enabled accurate identification of the different welding zones of dissimilar welds and presentation of clear results in all boundary zones. Colors indicate the zones with similar hardness values of the weld joint. The increase in hardness in the whole HAZ of AISI 420 was caused by the grain size reduction of martensite. Within a relatively short distance from the HAZ, the weld hardness rapidly passed to the base metal hardness level.

**Figure 12:**
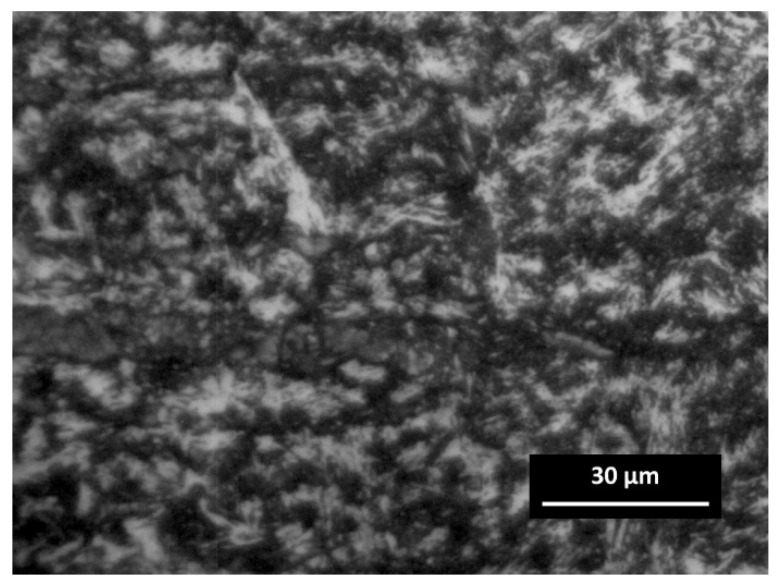
Optical image of untempered martensite in the HAZ of AISI 420.

The martensite was formed when the HAZ close to the FZ was heated above the transformation temperature during welding. Usually, unwanted martensite is considered negative, and its formation indicates inadequate welding procedures. The coarse untempered martensite was observed in the HAZ (Figure 12), while the formation of fine martensitic microstructure was revealed in the BM as it is presented in Figure 8a. Moreover, as the temperature decreased at a distance from HAZ, the carbon diffusion decreased as well as the coarsening of precipitation.

Saturation of the martensitic base with a certain amount of carbon and nitrogen can also cause the increase in hardness in this area: the hardness values ranged from 200 to 505 HV/0.1. It has been reported that a martensitic structure of high hardness generally has a low fracture toughness and is considered as highly susceptible to hydrogen-induced cold cracking compared with structures of lower hardness and higher fracture toughness [29]. The lower hardness level of the BM, compared with that found in the HAZ, is associated and can be explained by the large grain size in the BM.

**Figure 13:**
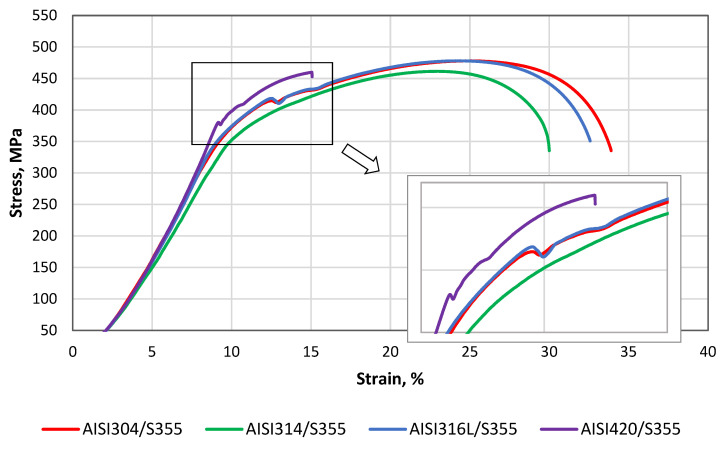
Stress–strain diagrams of dissimilar welds.

To assess the suitability of different grades of stainless steels for a particular application area and to evaluate the strength of dissimilar welds, the tensile tests were carried out. The tensile stress–strain behavior of samples S355MC/AISI 304, 314, 316L, and 420 is presented in Figure 13.

The tensile tests revealed the values of the tensile strength of the dissimilar welds, and, as can be seen in Figure 13, the values of AISI 304 and AISI 316L were very closely distributed (477.7 ± 2.5 and 478.0 ± 2.5, respectively), while the tensile strengths of AISI 314 and AISI 420 were slightly lower (461.4 ± 2.5 and 459.8 ± 2.5 MPa, respectively). Tensile tests of all austenitic stainless steels and the structural steel S355MC dissimilar weld confirmed the acceptable joint strength. The lower strength of AISI 420/S355MC led to the fracture at a significantly lower relative elongation compared to the other three weldments. Figure 13 clearly shows that the sample AISI 420/S355MC failed the tensile test. This behavior is explained by the mechanical properties of HAZ of AISI 420 weld side, in particular, ultimate tensile strength and yield strength, as well as by hardness tests’ results. Since the hardness of the HAZ area of AISI 420 was higher than the hardness of HAZ area of S355MC and of both base metals (Figure 9), the ductility parameters of the HAZ area of AISI 420 were significantly lower than those of the HAZ area of S355MC and both BMs’ due to increased hardness, brittleness, and a higher carbon content.

**Figure 14:**
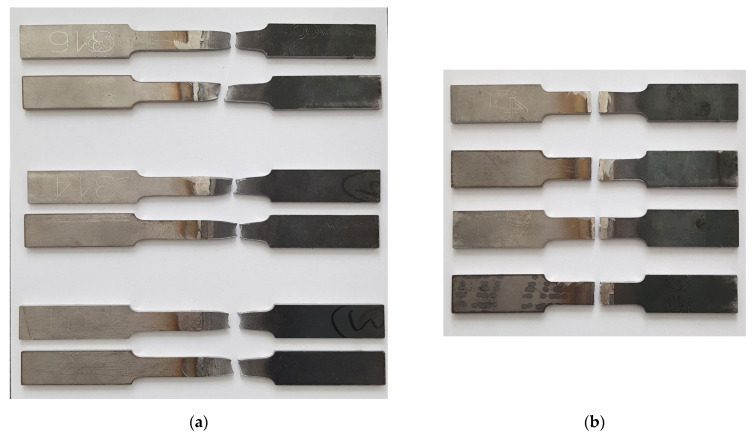
The fracture positions on tensile samples: (**a**) austenitic stainless steels/S355MC; (**b**) martensitic stainless steel/S355MC.

It can be clearly seen in Figure 14a that all cases of the dissimilar austenitic stainless steels AISI 304, 314, and 316L and the structural steel S355MC weld fracture occurred in the base metal S355MC that met the safety requirements for the dissimilar welds; however, when welding martensitic stainless steel AISI 420 and S355MC, a fracture occurred through the weld seam. All the samples of AISI 420 and S355MC dissimilar welds failed at the weld area without any significant necking (Figure 14b). The fracture in AISI 420 occurred between the PMZ and the BM weld zone from the AISI 420 part. The tensile tests showed lower tensile strength of these samples compared with AISI 304, 314, 316L, and S355 dissimilar welds.

**Figure 15:**
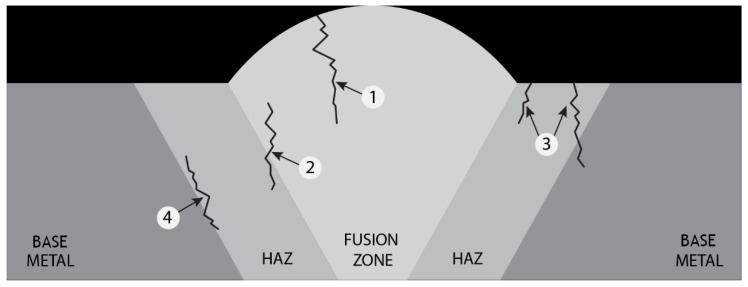
The classification of cracking in the weldments.

None of the tested samples of AISI 420 possessed fractures in the BM and in the FZ. The location of the fracture can also be explained by the fact that the higher strength was associated with the higher strength of untempered martensite, and this area was closer to the FZ (Figure 8). According to classification of crackings in weldments [30], there was a possible defect (No. 4) (Figure 15), which formed at the outer edge of the fine-grained HAZ close to the BM in the over-tempered region.

This type of cracking forms because of HAZ and weld differences in the carbon activity, which is concerned with different concentrations of Cr [30]. The mechanism of cracking for No. 4 is typical for welded steels with a chromium content of 9 to 14 percent [31], in case of AISI 420—13.68% (Table 1).

Moreover, the grain size in the weld area was different from that of the base metal. This difference in grain sizes leads to the different yield stresses such that strength decreased (the Hall–Petch relationship [32]). The creep-like mechanism manages the fracture in this zone. Obviously, the formation of this mechanism, which is limited specifically to the fined-grained regions have to be avoided.

## 4. Conclusions

Summarizing the results of the performed detailed research, it can be stated that MIG welding using the filler rod 308LSi is suitable to produce dissimilar 3xx and 4xx series steel to structural steel welds. However, the results of the studies showed that the combination of martensitic steel and structural steel lags behind the stainless steel in the tensile test. This is explained by the formation of untempered martensite in the HAZ (~500 HV/0.1) due to the high carbon content (0.441%) in the alloy. Since PWHT was not used in order to study dissimilar welds under the same conditions, a lower tensile strength of 459.8 ± 2.5 MPa for the martensitic-structural steel weld was achieved because of the disparity in grain sizes, which greatly affects the reduction in strength. The fracture in the weld seam zone occurred from the martensitic stainless-steel part in the HAZ. The predominantly untempered martensitic structure resulted in low ductility and a hard and brittle HAZ area that fractured before necking with a negligible elongation. More promising results were obtained from the study of austenitic stainless-structural steel welds. The fracture in all 3xx series and structural steel welds occurred in the structural steel zone far from the fusion zone, where a very similar tendency with a slightly higher hardness of the stainless steel base ~185 HV/0.1 than of the structural steel base ~167 HV/0.1 was achieved. Similar results were achieved while executing tensile tests of 3xx series and structural steel welds: the same tendency of stress–strain curves had a 472 MPa tensile strength on average. It is concluded that martensitic and structural steel welds without PWHT are characterized by brittle fractures, and therefore this type of joint cannot be used in critical applications safely.

## Figures and Tables

**Table 1:** Chemical composition of welded sheets.

Grade	Chemical Composition (%)									
	C	Mn	Si	P	S	Cr	Ni	Cu	N	Mo
AISI 304	0.027	1.780	0.320	0.030	0.0010	18.05	8.05	-	0.660	-
AISI 314	0.056	1.520	1.810	0.022	0.0010	24.19	19.06	0.180	0.051	-
AISI 316L	0.020	1.210	0.570	0.031	0.0010	16.80	10.10	-	-	2.10
AISI 420	0.441	0.280	0.360	0.027	0.0013	13.68	-	-	0.040	-
S355MC	0.049	0.789	0.012	0.015	0.0080	-	-	-	-	-
ER 308LSi *	0.017	1.890	0.740	0.017	0.0110	19.67	10.20	0.120	0.074	0.16
Fe–balance										

* Filler road.

**Table 2:** Mechanical properties of steels.

Grade	Mechanical Properties					
	Yield Point *R_p_*_(*0.2*)_ (MPa)	Yield Point *R_p_*_(*1*)_ (MPa)	Tensile Strength *R_m_* (MPa)	Elongation (%)	Young’s Modulus (GPa)	Hardness (HV)
AISI 304	288	333	609	59.3	190–203	167
AISI 314	309	360	609	56	200	176
AISI 316L	286	290	608	57.5	190–205	163
AISI 420	345	345	636	26	200	180
S355MC	392	392	452	41	190–210	155–195
ER 308LSi *	≥320		≥510	≥25		160

* Filler road.

**Table 3:** The main parameters of welding procedure.

Welding Mode	MIG Current (A)	MIG Voltage (V)	Wire Feed Speed (m/s)	Pulse Duration during Welding (s)	Heat Input (J/mm)
SuperPuls (max)	74	19.5	7.2	0.15 (74A)	230.88
SuperPuls (min)	40	16.2	4.0	0.20 (40A)	103.68
Impulse	74	19.5	7.2	-	230.88
Travel speed 5.0 mm/s Factor of thermal efficiency (MIG) 0.8					

## Data Availability

Data sharing not applicable.
